# Supplementary material for: Natural Products as Inducers of Non-Canonical Cell Death: A Weapon against Cancer
Source: Cancers (Basel). 2021 Jan 15;13(2):304. doi: 10.3390/cancers13020304 (PMC7830727; doi:10.3390/cancers13020304)
Supplement: Supplementary file 1 [file cancers-13-00304-s001.pdf]

Supplementary Materials

Table S1. In vitro induction of ferroptosis by natural products used in association.

Compound	Compound source	Cell line(s)	Concentration range (where specified)	Time (where specified)	Ferroptosis markers	Supplementary effects	Reference
**Albiziabioside A (AlbA) conjugated with dichloroacetate (DCA)**	*Albizia inundata* Mart.	MCF-7	2 μM	24h	↑ Cytotoxicity	↑ after Fe^{2+} treatment<br>↓ after Ferr-1 treatment<br>↓ after DFO treatment<br>↓ after vitamin E treatment	[24]
				/	↑ ROS		
				24h	↓ GSH/GSSG *ratio*		
				48h	↓ GPX4 expression		
				/	↑ MDA<br>↑ Lipid peroxides		
**Gallic Acid (GA) + low level of laser irradiation**		A375, MDA-MB-231	10, 25, 50, 100 and 200 μg/mL	/	↓ Cell viability		[44]
		MDA-MB-231	25 μg/mL	24h	↑ ROS		
		A375	50 μg/mL				
		A375, MDA-MB-231	/	/	↑ MDA<br>↓ GPX4 activity		
**Phenethyl isothiocyanate (PEITC) + cotylenin-A (CN-A)**	Cruciferous vegetables	MIAPaCa-2	PEITC (5 μM) ± CN-A (10 μg/mL)	12 days	↓ Anchorage-independent growth		[243]
		MIAPaCa-2, Panc-1	PEITC (6 μM) ± CN-A (15 μg/mL)	16h	↓ Cell viability	↑ after NAC treatment<br>↑ after Trolox treatment	

			PEITC (5 μM) ± CN-A (20 μg/mL)	24h	↑ Cell death		
		Panc-1	PEITC (2, 4, 6 and 8 μM) ± CN-A (15 μg/mL)	16h	↑ Cell death	↓ after Ferr-1 treatment<br>↓ after Lip-1 treatment<br>↑ after diferric holotransferrin treatment	
		MIAPaCa-2, Panc-1	PEITC (4 μM) ± CN-A (20 μg/mL)	4h	↑ ROS		
**Piperlongumine (PL) + cotylenin A (CN-A)**	*Piper Longum* L.	Panc-1, MIAPaCa-2, CFPAC-1	PL (2, 4, 6, 8 and 10 μM) + CN-A (15 μg/mL)	16h	↓ Cell viability		[46]
		MIAPaCa-2	PL (6 μM) + CN-A (15 μg/mL)	16h	↓ Cell viability	↑ after NAC treatment<br>↑ after Ferr-1 treatment<br>↑ after Lip-1 treatment<br>↑ after DFO treatment	
		Panc-1	PL (6 μM) + CN-A (15 μg/mL)	16h	↓ Cell viability	↑ after NAC treatment<br>↑ after Ferr-1 treatment<br>↑ after Lip-1 treatment<br>↑ after DFO treatment<br>↑ after 3-MA treatment	
		MIAPaCa-2	PL (7.5 and 15 μM) ± CN-A (15 μg/mL)	4h	↑ ROS		
**Piperlongumine (PL) + sulfasalazine (SSZ)**	*Piper Longum* L.	Panc-1, MIAPaCa-2, CFPAC-1, BxPC-3	PL (2.5, 5 and 7.5 μM) ± SSZ (200 μM)	16h	↓ Cell viability		[46]
				16h	↓ Cell viability	↑ after NAC treatment	

		Panc-1, MIAPaCa-2	PL (5 μM) + SSZ (250 μM)			↑ after Ferr-1 treatment<br>↑ after DFO treatment	
		MIAPaCa-2	PL (7.5 and 15 μM) + SSZ (200 μM)	4h	↑ ROS		
**Piperlongumine (PL) + cotylenin A (CN-A) + sulfasalazine (SSZ)**	*Piper Longum* L.	PANC-1, MIAPaCa-2	PL (2, 4, 6 and 8 μM) + CN-A (15 μg/mL) + SSZ (250 μM)	16h	↓ Cell viability	↑ after Ferr-1 treatment	[46]

Abbreviations.

↑: Increase; ↓: Decrease; 3-MA: 3-Methyl-adenine; DFO: Deferoxamine; Fe^{2+}: Ferrous ion; Ferr-1: Ferrostatin 1; GPX4: Glutathione peroxidase 4; GSH: Glutathione; GSSG: Oxidized glutathione; Lip-1: Liproxastatin-1; MDA: Malondialdehyde; NAC: N-acetylcysteine; ROS: Reactive oxygen species.

Table S2. Natural products as *in vivo* inducers of ferroptosis.

Compound	Compound source	Experimental model(s)	Treatment doses	Antitumor effects and ferroptosis markers	Reference
**Amentoflavone**	*Selaginella* spp. and other plants	BALB/c nude mice inoculated with U251 cells	40 and 80 mg/kg/day (for 28 days)	↓ Tumor volume<br>↓ Tumor weight<br>↓ GSH<br>↑ MDA<br>↓ FTH<br>↑ LC3B, ↑ Beclin1, ↑ ATG5, ↑ ATG7	[25]
**Artenusate (artemisin semi-synthetic derivative)**	*Artemisia annua* L.	NOD/SCID mice inoculated with CA-46 cells	200 mg/kg/day (30 days)	↓ Tumor volume<br>↓ Tumor weight<br>↑ MDA<br>↑ CHAC1 expression	[29]
		BALB/c nude mice inoculated with AsPC-1 cells transfected with control (CTRL) or GRP78 shRNA	30 mg/kg every 2 days (35 days)	↓ Tumor growth (> than CTRL shRNA)<br>↓ Tumor volume (> than CTRL shRNA)<br>↓ Tumor weight (> than CTRL shRNA)	[32]
**Dihydroartemisinin (artemisin semi-synthetic derivative) (DAT)**	*Artemisia annua* L.	Foxn1nu/ Foxn1+ athymic mice inoculated with GPX4 iKO H292 cells	DAT 5 mg/kg/day, normal diet/DAT 5 mg/kg/day, DOX diet / DAT 5 mg/kg/day + DOX and Lip-1 10 mg/kg (14 days)	↓ Tumor growth (partially restored after Lip-1 treatment)	[63]
**Withaferin A**	*Withania somnifera* L. Dunal	BALB/c nude mice inoculated with IMR-32 cells	Bi-sequential treatment (4 days treatment with 4 mg/kg/day + 6 days of recovery + 3 days treatment with 4 mg/kg/day);	↓ Tumor growth<br>↓ Tumor relapse rates<br>CD45-positive immune cells infiltration in tumor tissue<br>↓ GPX4 expression	[52]

	continuous treatment (4 mg/kg/day, for 20 days)	↑ LOOH
		↑ phospolipids (phosphatidylglicerol/phosphatidylinositol)
	WA-NPs (days 1 and 5, 14 days)	↓ Tumor growth

Abbreviations.

↑: Increase; ↓: Decrease ATG5: Autophagy related 5; ATG7: Autophagy related 7; CHAC1: Glutathione-specific gamma-glutamylcyclotransferase 1; DOX: Doxorubicin; FTH: Ferritin heavy chain; GPX4: Glutathione peroxidase IV; GSH: Glutathione; LC3B: Microtubule-associated protein 1 (MAP1) light chain 3B; Lip-1: Liproxastatin-1; LOOH: Lipid hydroperoxides; MDA: Malondialdehyde; Spp.: species; WA-NPs: Withaferin A nanoparticles

Table S3. *In vitro* induction of necroptosis by natural products used in association.

Compound	Compound source	Cell line(s)	Concentrations (where specified)	Time (where specified)	Necroptosis markers	Supplementary effects	Reference
**Arctigenin (A) + desamethasone (D)**	*Arctium lappa* L., *Saussurea heteromalla*	RPMI-2650	A (5 μM) + D (12.5 μM)	48h	↑ Necrotic cells	↓ after NAC treatment	[114]
					↑ ROS	↓ after NAC treatment	
					↓ ΔΨm	↑ after NAC treatment	
					↓ ATP levels	↓ after Nec-1 treatment	
					↑ RIP3, ↑ p-RIP3 protein expression	↓ after Nec-1 treatment	
						↓ after ATP treatment	
						↓ after NAC treatment	
					↑ MLKL, ↑ p-MLKL protein expression	↓ after Nec-1 treatment	
						↓ after ATP treatment	
						↓ after NAC treatment	
					↑ p-ATM, ↑ p-ATR, ↑ p-CHK1/2 protein expression	↓ after NAC treatment	
				24, 48 and 72h	↓ Cell viability	↑ after Nec-1 treatment	
						↑ after ATP treatment	
						↑ after NAC treatment	
		RPMI-2650 spheroid cells	A (5 μM) + D (12.5 μM)	48h	↑ Cytotoxicity	↓ after NAC treatment	
					↓ Spheroid viability	↑ after NAC treatment	
					↑ RIP3, ↑ p-RIP3 protein expression	↓ after NAC treatment	
					↑ MLKL, ↑ p-MLKL protein expression	↓ after NAC treatment	
**Berberine (B) + cisplatin (Cis)**	Huang Lian Chinese herb (*Coptis chinesis*) and others plant spp.	OVCAR3	B (100 μM) + Cis (5 mg/mL)	24h	Extensive vacuolation		[116]
					Rupture of plasma membrane		
		OVCAR3, POCCLs			↑ RIP3, ↑ MLKL mRNA levels		
					↑ RIP3, p-RIP3 protein expression		
					↑ MLKL, p-MLKL protein expression		

**Goniothalamin + Z-VAD-FMK 10 μM**	Plants of *Goniothalamus* genus	MDA-MB-231	33.82 μM	24h	↑ PI positive cells		[244]
					Loss of membrane integrity		
					No caspase-3/-8/-9 activation		
					↑ Calpain activity	↓ after Z-LLY-FMK treatment	
			13.53 and 33.82 μM	1, 2, 4 and 6h	↑ ROS	↓ after NAC treatment	
				24h	↑ Ca^{2+} levels		
					↑ RIP3 protein expression		
					↑ Cleaved AIF		

Abbreviations.

↑: Increase; ↓: Decrease; AIF: Apoptosis-inducing factor; ATP: Adenosine triphosphate; Ca^{2+}: Calcium; MLKL: Mixed lineage kinase domain like pseudokinase; NAC: N-acetyl-L-cysteine; Nec-1: Necrostatin-1; p-ATM: Phospho-ataxia telangiectasia mutated kinase; p-ATR: Phospho-ataxia telangiectasia and Rad3-related kinase; p-CHK1/2: Phospho-checkpoint kinase 1/2; p-MKLK: Phospho-mixed lineage kinase domain like pseudokinase; p-RIP3:Phospho-receptor-interacting serine/threonine-protein kinase 3; PI: Propidium iodide; RIP3: Receptor-interacting serine/threonine-protein kinase 3; ROS: Reactive oxygen species; Spp.: species; Z-LLY-FMK: Calpain inhibitor; $\Delta\Psi m$: Mitochondrial membrane potential.

Table S4: Natural products as *in vivo* inducers of necroptosis.

Compound	Compound source	Experimental model(s)	Treatment doses	Necroptosis markers	Reference
**2-methoxy-6-acetyl-7-methyljuglone**	*Polygonum cuspidatum* Sieb. et Zucc	U251-derived xenograft zebrafish model	50 nM (3 days)	No caspase- 3/-7/-8 cleavage	[108]
				↑ p-JNK1/2	
				↑ HMGB1	
		BALB/c nude mice inoculated with A549 cells	1 and 2 mg/kg/day (18 days)	↓ Tumor growth	[109]
				↓ Tumor volume	
				↓ Tumor weight	
				↑ RIP1 protein expression	
				↑ p-RIP3 protein expression	
				Intact cell nuclei	
				Damaged mitochondria	
**Emodin**	*Rheum palmatum* L.	BALB/c nude mice inoculated with U251 cells	20, 40 and 80 mg/kg/day (28 days)	↓ Tumor growth	[121]
				↓ Tumor volume	
				↓ Tumor weight	
				↑ RIP1 protein expression	
				↑ RIP3 protein expression	
				↑ MLKL protein expression	
				↑ TNF-α protein expression	
				↑ Tumor necrosis	
**Pristimerin**	Various plant spp. of *Celastraceae* and *Hippocrateaceae* families	BALB/c nude mice inoculated with C6 cells	1 and 3 mg/kg every 2 days (8 days)	↓ Tumor volume	[127]
				↓ Tumor weight	
				↑ JNK protein expression	
				↑ p-JNK protein expression	
				↑ AIF nuclear translocation	
**Resibufogenin**	Asiatic toad (*Bufo gargarizans*)	BALB/c nude mice inoculated with eGFP-SW480 cells	5 and 10 mg/kg/day (21 days)	↓ Tumor volume (partially restored after RIP3 knockdown)	[129]
				↓ Tumor weight	

				(partially restored after RIP3 knockdown)	
				↑ RIP3 protein expression	
				↑ PYGL, ↑ GLUL, ↑ GLUD1 protein expression	
		C57BL6/j mice inoculated with eGFP-MC38 cells		↓ Number of liver metastatic foci	
				↓ Size of liver metastatic foci	
**Shikonin**	*Lithospermum erythrorhizon* Siebold & Zucc., *Arnebia euchroma* (Royle) Johnst, or *Arnebia guttata* Bunge	Nude mice inoculated with CNE-2Z cells	0.5 and 1 mg/kg every 3 days (21 days)	↓ Tumor growth	[133]
				↓ Tumor weight	
				↑ Tumor necrosis	
		BALB/c athymic nude mice inoculated with A549 cells	2 mg/kg/day (14 days)	↓ Tumor growth	[134]
				↓ Tumor volume	
				↓ Tumor weight	
				↑ Tumor necrosis	
				↑ RIP1 protein expression	
		BALB/c nude mice inoculated with K7 cells	2 mg/kg/day (7 days)	↓ Tumor growth	[136]
				↓ Tumor volume	
				↓ Tumor weight	
				↑ RIP1 protein expression	
				↑ RIP3 protein expression	
				↓ Number of lung metastasis	
				↓ Lung weight	
				↑ Tumor necrosis in lung metastasis	
		BALB/c nude mice inoculated with C6 cells	2 mg/kg every 2 days for 4 times	↓ Tumor volume	[140]
				↓ Tumor weight	
				↑ RIP1 protein expression	
				↑ RIP3 protein expression	
				↑ γ-H2AX protein expression	
				↑ p-ATM protein expression	

			RIP1-RIP3 interaction	
			↑ HMGB1 protein expression	
			↑ HSP70 protein expression	
			↑ Calreticulin protein expression	
			Macrophages infiltration in tumor tissue	
			↓ Tumor volume	[141]
			↑ Cytoplasmatic/mitochondrial p-MLKL protein expression	
			↓ Mitochondrial AIF protein expression	
			↑ Cytoplasmatic/nuclear AIF protein expression	
	BALB/c nude mice inoculated with 5-8F cells	0.5 mg/kg every 3 days (21 days)	↓ Tumor growth	[142]
			↓ Tumor weight	
			↑ Tumor necrosis	

Abbreviations.

↑: Increase; ↓: Decrease; AIF: Apoptosis-inducing factor; GLUD1: Glutamate dehydrogenase; GLUL: Glutamine synthetase; HMGB1: High-mobility group box 1; HSP70: Heat shock protein 70; JNK: c-Jun-N-terminal kinase; MLKL: Mixed lineage kinase domain like pseudokinase; p-ATM: Phospho-ataxia telangiectasia mutated kinase; p-JNK1/2: Phospho-c-Jun N-terminal kinase 1/2; p-MLKL: Phospho-mixed lineage kinase domain like pseudokinase; p-RIP3: Phospho-receptor-interacting serine/threonine-protein kinase 3; PYGL: Glycogen phosphorylase; RIP1: Receptor-interacting serine/threonine-protein kinase 1; RIP3: Receptor-interacting serine/threonine-protein kinase 3; TNF-α: Tumor necrosis factor-α; γ-H2AX: Phospho-H2A histone family member X.

Table S5. Natural products as *in vivo* inducers of pyroptosis

Compound	Compound source	Experimental model(s)	Treatment doses	Antitumor effects and pyroptosis markers	Reference
**Alpinumisoflavone**	*Derris eriocarpa* F.C	BALB/c nude mice inoculated with KYSE30 cells	40 and 80 mg/kg/day (30 days)	↓ Tumor volume	[198]
				↓ Tumor weight	
				↑ GSDME, ↑ cleaved caspase-3, ↑ Bax expression	
		BALB/c nude mice inoculated with TPC-1 cells	20 and 40 mg/kg/day (30 days)	↓ Tumor volume	[199]
				↓ Tumor weight	
				↑ NLRP3, ↑ cleaved caspase-1, ↑ cleaved IL-1β, ↑ cleaved IL-18, ↑ cleaved GSDMD protein expression	
**Berberine**	Huang Lian Chinese herb *(Coptis chinesis)* and others plant spp.	BALB/c-nude mice inoculated with HepG2 cells	20 mg/kg/day (21 days)	↓ Tumor volume (partially restored after AC-YVAD-CMK treatment)	[201]
				↑ Caspase-1 mRNA and protein expression (↓ after AC-YVAD-CMK treatment)	
**Dioscin**	*Polygonatum zanlanscianense* Pamp., *Dioscorea nipponica* Makino, and *Dioscorea zingiberensis* C. H. Wright	BALB/c mice inoculated with MNNG/HOS cells	12 and 24 mg/kg, every 3 days (15 days)	↓ Tumor volume	[203]
				↓ Tumor weight	
				↑ GSDME-NT expression	
				↑ Cleaved caspase-3 expression	
**Huaier extract**	*Trametes robiniophila* murr (Huaier)	BALB/c nude mice inoculated with H520 cells	50 mg/mL/day (28 days)	↓ Tumor volume	[205]
				↑ NLRP3, ↑ cleaved-caspase-1, ↑ IL-1β, ↑ IL-18 protein levels in tumor tissue	
**Polyphyllin VI**	*Trillium tschonoskii* Maxim	Athymic nude mice inoculated with A549 cells	2.5, 5 and 10 mg/kg/day (10 days)	↑ NLRP3, ↑ cleaved caspase-1, ↑ cleaved IL-1β, ↑ cleaved GSDMD protein levels	[195]
				↑ NLRP3, ↑ caspase-1, ↑ IL-1β, ↑ GSDMD protein levels in tumor tissue	

Abbreviations.

↑: Increase; ↓: Decrease; AC-YVAD-CMK: Caspase-1 inhibitor; GSDMD: Gasdermin D; GSDME: Gasdermin E; GSDME-NT: N terminal fragment of Gasdermin E; IL-18: Interleukin-18; IL-1β: Interleukin 1 beta; NLRP3: NLR (nucleotide-binding oligomerization domain (NOD)-like receptor) family pyrin domain-containing 3; Spp.: species
